# On the way to plant data commons – a genotyping use case

**DOI:** 10.1515/jib-2022-0033

**Published:** 2022-09-05

**Authors:** Manuel Feser, Patrick König, Anne Fiebig, Daniel Arend, Matthias Lange, Uwe Scholz

**Affiliations:** Leibniz Institute of Plant Genetics and Crop Plant Research (IPK) Gatersleben, 06466 Seeland, Germany

**Keywords:** biodiversity, cloud computing, imputation, plants, research data commons

## Abstract

Over the last years it has been observed that the progress in data collection in life science has created increasing demand and opportunities for advanced bioinformatics. This includes data management as well as the individual data analysis and often covers the entire data life cycle. A variety of tools have been developed to store, share, or reuse the data produced in the different domains such as genotyping. Especially imputation, as a subfield of genotyping, requires good Research Data Management (RDM) strategies to enable use and re-use of genotypic data. To aim for sustainable software, it is necessary to develop tools and surrounding ecosystems, which are reusable and maintainable. Reusability in the context of streamlined tools can e.g. be achieved by standardizing the input and output of the different tools and adapting to open and broadly used file formats. By using such established file formats, the tools can also be connected with others, improving the overall interoperability of the software. Finally, it is important to build strong communities that maintain the tools by developing and contributing new features and maintenance updates. In this article, concepts for this will be presented for an imputation service.

## 1 Introduction

The volume of data with high potential for exploitation in research in general and in plant research in particular has increased due to technological advancements in genomic analysis, precision phenotyping, and digitalization in the scientific value chain [1]. Data is captured, processed and stored by a variety of stakeholders, with different primary interests in various data flow scenarios. The types of data generated range from temporal and spatial data on quantitative and qualitative traits, molecular characteristics, to records of field experiments that may include information on fertilization, crop protection, field and soil conditions, or weather data, in addition to agronomic and breeding traits. Data differ not only in their subject of study, but also in their type, format, and context of origin.

This article highlights current challenges and possible solution strategies in plant genotyping using the example of the Leibniz Institute of Plant Genetics and Crop Plant Research (IPK) Gatersleben. The transformation of genebanks into bio-digital resource centers [2] is a process that involves many challenges and requires an alliance of information technology, standardization, sustainable Research Data Management processes [3] as well as a high degree of national and international networking in the technical and infrastructure communities. Thus, the IPK participates with infrastructures, databases, training programs and human capacities in infrastructure programs such as NFDI [4], GFBio [5], de.NBI [6], ELIXIR [7], ECPGR [8], DivSeek [9]. Concrete contributed RDM related services include the institutionally supported genebank information system “GBIS/I” [10] or the data publication service “Plant Genomics & Phenomics Research Data Repository – e!DAL-PGP” for DOI-based publication of plant research data [10–12]. In addition, the IPK takes an active role in the development of standards. Examples include membership in ORCID-DE, DataCite, and contributions to standardization initiatives such as the Breeding API [13, 14], or the harmonization of formats for storing sequence and diversity data such as proposed by the FONDUE project [15, 16].

The advantages and effectiveness of this modular approach have been demonstrated by already finished and still ongoing research projects in the field of plant genetic resources. These include the creation of a homogeneous data space of plant genetic resources stored in genebanks and their web-based interactive exploration [17], the digital integration of European genebanks to store plant genetic resources (PGR) [18], or the establishment of common RDM structures to access plant phenotyping data [19].

Especially genotyping and imputation as a subfield in particular, highly depends on the quality of the underlying RDM. So, based on the experience from these projects, an eye is cast on a current workflow for imputation of genotypic data. In particular, data for imputation is currently stored locally and processed sequentially on virtual machines. With a transition to a cloud-based, distributed solution, the efficiency as well as sustainability is expected to be increased significantly. In the following, the individual services involved are described, before explaining the current workflow in detail. Afterwards, suggestions are made to modernize the current imputation services as well as discussing the benefits and newly arisen challenges. This is enabled and accompanied by the modular approach and the specification of well-defined interfaces as well as the usage of standardized formats. Finally, it is shown how further synergies can be generated through integration into international as well as national programs such as the National Research Data Infrastructure (NFDI). In particular, the IPK is involved in the consortium for biodiversity research NFDI4Biodiversity, where one goal is to establish a cloud computing infrastructure with the Research Data Commons (RDC) to provide analysis services.

## 2 Material and methods

IPK’s RDM strategy aims to support a federated scenario of research groups under the umbrella of a central service portfolio to support all steps of the RDM data life cycle [3, 20, 21].

**Figure 1:**
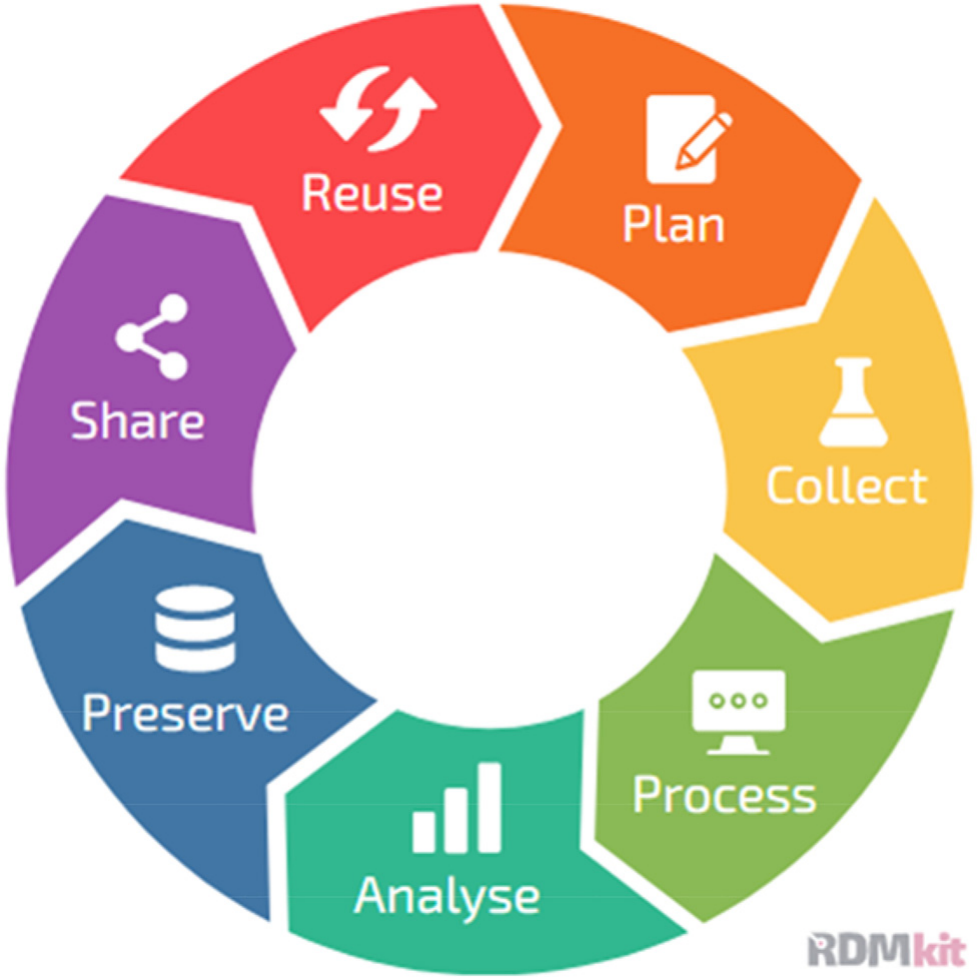
Taken from ELIXIR (2021) research data management kit. A deliverable from the EU-funded ELIXIR-CONVERGEproject (grant agreement 871075) [WWW Document]. https://rdmkit.elixireurope.org. Accessed 25 May 2022.

The seven steps of the research data lifecycle (Figure 1) are fulfilled by

1. the Research Data Management Organiser (RDMO) [22] as a questionnaire-based tool for creating project-specific data management plans,
2. the LIMS to support data collection in the labs, greenhouses and in the field,
3. the data pre-processing according to common guidelines and
4. the provisioning of staging areas, a Galaxy instance to stage commonly used data analysis pipelines and a Slurm cluster for individual software pipelines,
5. sharing through an ORACLE Application Express [23] for declarative development of web portals,
6. data sharing through data steward-based data publishing pipelines in central repositories, such as EMBL-ENA, EMBL-EVA or e!DAL-PGP [11], and last but not least
7. the collaboration and dissemination of international standards for data and metadata, such as MIAPPE [24], BioSchemas [25], MCPD [26] or BioSamples [27].

In this section, the focus will be on the three steps Store, Share and (Re)Use of the data lifecycle. The selection refers to outcomes from various projects with IPK participation that are connected to the genotyping domain and are related to the use case of imputation. However, the scope could be extended further, but will be limited for the purposes of this article to the aspect of released or ongoing developments from IPK or community developed outcomes with IPK contribution.

### 2.1 Store

As a generic data management backend, a stack of a highly available commercial relational database management system (RDBMS) and a file storage appliance, a hierarchical storage management system (HSM), is operated by core funded IT to host all IPK research data.

The actual implementation of project specific data structures is in majority brokered by an institute-wide available laboratory information management system (LIMS) for structured data and an electronic laboratory book (ELN) for the documentation of experimental work [20]. The common technical foundation is the central RDBMS together with the HSM. In order to perform a data integration, data curation needs to accompany all phases of data ingestion. This means, in particular, that uniform material identifiers, homogeneous plant material descriptions and uniform measurement scales, such as common scoring schemes, are used [21]. This work is coordinated by LIMS managers and carried out in consultation with the respective domain experts.

The European Variation Archive (EVA) [28], which is part of the European Bioinformatics Institute (EMBL-EBI) infrastructure supports all types of genetic variation data from all species, except privacy-restricted datasets like from humans. Data is available via EVA’s study and variant browser or can be retrieved through FTP or API. A use case for an FAIR compliant submission of plant genotyping datasets has been described lately [27].

A submission to EBI-EVA requires a valid Variant Call Format (VCF) [16] file that is based on a reference sequence available to any INSDC [29] repository (EMBL, NCBI or DDBJ) and a template-based Microsoft Excel sheet describing study metadata and – if not made available beforehand – sample metadata. To avoid delays, the VCF files should be checked for a correct syntax using validator tools provided by the EBI-EVA. The following semi-automatic submission might become a lengthy procedure especially in case of large VCF files but ensures high quality data publication.

The recommended workflow for a FAIR compliant genotyping dataset submission: [if not yet available: (0) Submit reference assembly to INSDC public repository], (1) Registering sample metadata at EBI-BioSamples, (2) Submission of raw read files at EMBL-ENA by using previously registered Biosamples records, (3) Prepare, validate and submit VCF file; previous ENA run IDs obtained from step 2 should be linked to the EVA study; BioSample IDs from step 1 are re-used as genotype names.

### 2.2 Share

One option to share data is by using files in established file formats. For genotypic diversity data, the Variant Call Format (VCF) [30] allows the storage of large sets of nucleotide-level variation such as Single Nucleotide Polymorphisms (SNPs) and also structural variations such as INDELs (insertions and deletions), inversions, copy number variations or translocations. A VCF file comprises a header part describing general metadata and the samples of the genotyping study and the body section that stores the actual sequence variations. Although VCF is the widely accepted standard format for storing and exchanging variant data, there is no explicit specification for study and sample metadata description. To overcome this limitation, a proposal [16] suggesting minimal metadata standards adapting FAIR (Findable – Accessible – Interoperable – Reusable) data principles has been released. To make use of these recommendations the use of the latest VCF specification v4.3 [31] is required.

In addition to the above-mentioned sharing of data by using files, the same can also be fulfilled via domain-specific APIs. The Breeding API (BrAPI) [14] is a community-driven RESTful-API specification for web services, that aims to support the access, exchange and integration of all data related to plant research. It is developed in a modular way by providing modules for the different data domains of plant breeding. One of them is the genotyping module, which was previously based on the data model of the GA4GH Variant Schema. In an attempt to optimize this module, it is currently extended to be more efficient, when requesting slices of a variant call matrix through a new endpoint. The new BrAPI version is available as version 2.1.

### 2.3 (Re-)Use

To analyze the diversity of various genotypes the method imputation is the attempt of completing sparse SNP data. The concept is based on the idea that unrelated samples in small regions are identical by descent (IBD). The differences between samples can be attributed to recombination of the originally underlying chromosomes. The goal of an imputation is now to identify those original haplotypes of the target haplotypes from a reference panel and to fill the gaps of the samples with the markers of the reference. In the following, reference markers are understood to be those markers that occur in the reference panel. Similarly, target markers are those from the target set and imputed markers are those that are not set in the target set and are taken from the reference panel [32]. Target markers need to be a subset of the reference markers.

Imputation enables and enhances several variant data analysis methods. For example, it enables the prediction of missing data, which has a positive impact on data analysis of only variant data. However, imputation also strengthens the expressiveness of inter-domain analysis methods, such as Genome Wide Association Studies (GWAS). It has been shown that the use of imputed data gives a 10% better result than an GWAS analysis on the raw data [33]. Imputation also allows the intersection of two different data sets, for example generated from different SNP arrays or genotyping by sequencing (GBS) data. Here, the target samples are merged on the basis of a common reference panel.

Various algorithms are available to perform imputation. Most of them are based on Hidden Markov Models (HMM), but also on positional Burrows-Wheeler transformation or SNP-tagging approaches. HMM approaches have better accuracy, since the whole chromosome, as well as all mosaic configurations of the haplotype are considered. Among the most used methods are IMPUTE [34], FastPHASE [35], Minimac [36] and Beagle [37]. Beagle seems to perform best in terms of computational complexity, considering time and space consumption, while preserving comparable error rates [32, 38].

To visualize and analyze the genotypic diversity data before and after the imputation, an inhouse developed web tool called DivBrowse [39] is used. It is written in Python and Javascript and is able to directly use VCF files to serve a web application for interactive visualization and analysis of the variant call matrices. Users are able to inspect the results of multiple imputation runs and compare them with each other on a nucleotide level. It can also act as a BrAPI endpoint for the imputation service so that the user does not have to upload the underlying VCF file to the imputation service first. Instead, the imputation service can directly load the complete diversity matrix or slices of it from a DivBrowse instance and start the process of imputation.

## 3 Results

### 3.1 Data import

**Figure 2:**
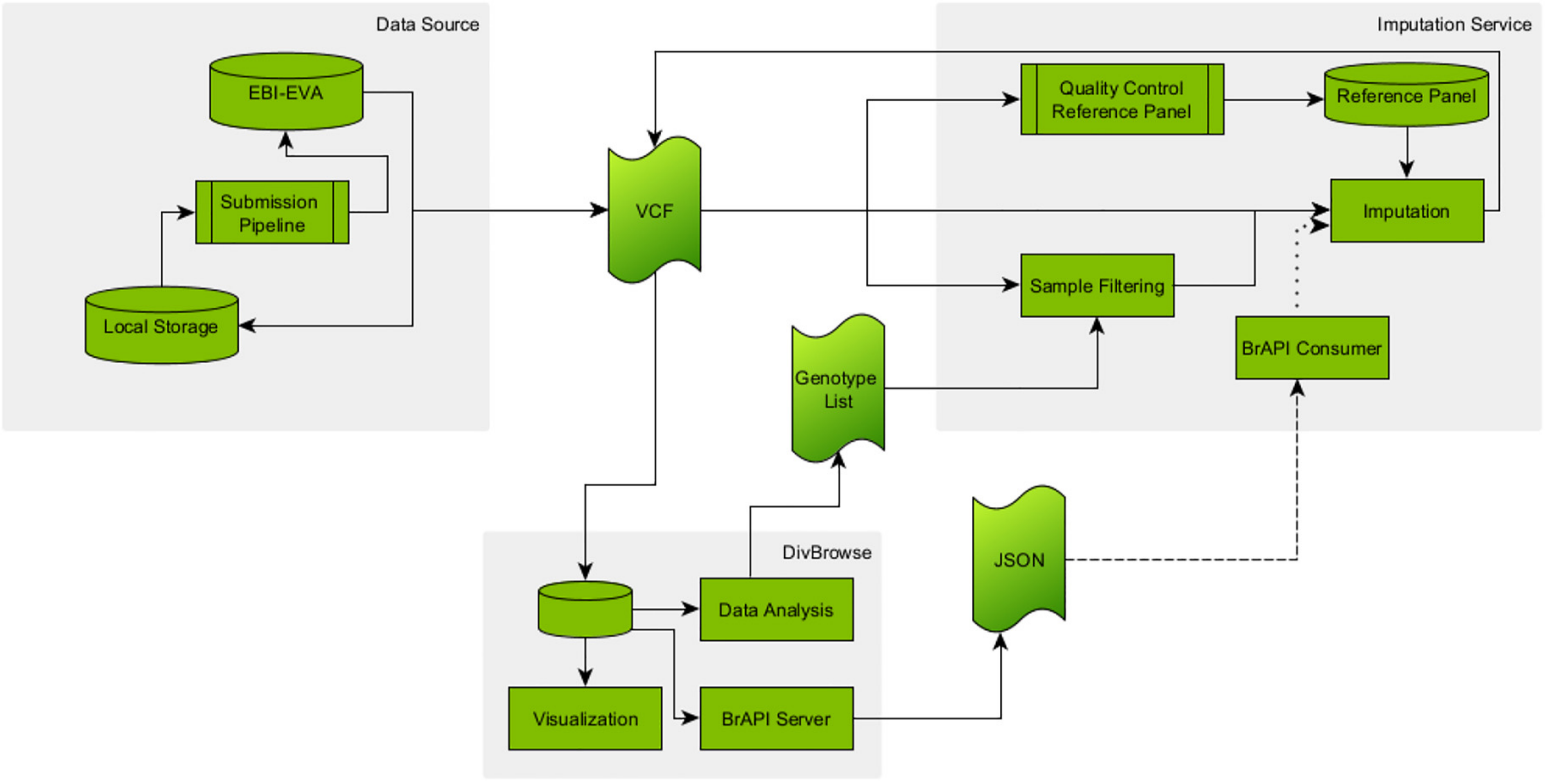
Depiction of the extended imputation workflow. Data from either public or local databases are provided as VCF files. DivBrowse can be used to perform data analysis for sample filtering, to make the data available via BrAPI or to visualize it. The imputation service includes the actual imputation, the filtering of VCF files, the pre-processing of reference panels and the planned processing of variant matrices from BrAPI endpoints. Solid line: Exists and used; Dashed line: Exists, but not used; Dotted line: Not existing.

The workflow for the imputation service, as depicted in Figure 2, starts with the data retrieval from one of the available data sources for variant datasets. The VCF file, or its binary counterpart BCF (binary variant call format) File, is either directly downloaded from the data source or copied from the local machine to the virtual machine running the imputation software, in this case Beagle. Eventually, one can decide to use a DivBrowse instance if already existing for the data set or set up a new one locally. This allows for a visualization as well as interactive analysis by PCA and UMAP of the sparse data set. The data analysis provides a lasso selection allowing one to create a list of samples. The original VCF file can afterwards be filtered on this list using some VCF manipulation software like bcftools and the new version of the file is finally sent to the imputation service.

### 3.2 Imputation

Two different options are offered for the imputation service. On the one hand, it is possible to impute only on the target set, on the other hand via a reference panel. The reference panel are dense variant calls that are used to fill the gaps in the target set. Here it is important that there is an intersection between the markers in the reference panel and the targets. For the reference panel, a sequence of steps is provided as a script for a minimal quality control, which is based on steps suggested in [40]. This ensures that (1) the naming conventions are properly followed and conforms the chromosome naming to the uniform format chrXX. Then (2) the rare variants, with allele count of one, are removed. It is also ensured that the VCF (3) contains only SNP data, before it is (4) aligned to a reference genome, which must be passed next to the VCF file, in order to bring the markers into the shortest representation. Subsequently, (5) all duplicates are removed before (6) allele frequencies and allele counts are recalculated if necessary. Finally, the (7) reference panel is converted to the binary reference panel file format bref and a (8) list of samples is created.

The preferred process is the imputation on a reference panel. To ensure that the chromosome, position and reference as well as allele nucleotides are identical, an additional program is provided. If there is no reference panel available the Beagle software can be run without one, imputing the missing data from the other samples of the target set.

### 3.3 Test run results

The described workflow was tested on a dataset to obtain a runtime comparable to the proposed improvements. For this test, a dataset consisting of 8070 wheat samples with a total of over 1.6 M unphased variants was used. The data set is submitted to the EMBL-EVA repository with the project identifier PRJEB52759. The imputation was performed on this data on a virtual machine in the de.NBI Cloud [41] that has 14 Intel Xeon Processors as VCPUs, 128 GB RAM, 20 GB disk as well as 2 TB of further volume storage. On this machine Beagle 4.1, bref in the associated version, as well as bcftools 1.10 were installed. The calculation ran in total more than 437 h (cf. Table 1 for more details on the analysis).

**Table 1:** Table with Run times for the Test Run on a single node over the complete dataset. Test run was executed on one virtual machine with 14 VCPU and 128 GB RAM.

	Total time for building model	Total time for sampling	Total run time
Single process run	92:53:58	338:16:33	437:18:38

### 3.4 Subsequent use of imputed data

Following imputation, the resulting VCF can be sent back to the local machine, so that it is available for further processing. Possible uses have already been mentioned in the previous section and, as far as they exceed the domain of genotypic data, will not be discussed further. A direct use case solely on the imputed data would be to load the resulting VCF file into a DivBrowse instance for visualization and analysis.

## 4 Discussion and outlook

To enable the best user experience for a cloud based imputation service, we implemented a handy data import and optimised the performance using a distributed, cloud based execution pipeline. This improved on one hand, the user-friendliness, on the other hand, the runtimes of the individual imputations. The following sections outline additional possible future steps to be taken into those directions.

### 4.1 Improvements to data import

A potential hurdle in usability is that the imputation service runs on a virtual machine that is controlled via the command line. The user then executes a number of scripts in a predefined order with individualized parameters, thereby semi-automatically managing the jobs. This whole process can be simplified by providing a web interface. Such an interface should allow the user to (1) submit the target data, (2) change the reference, (3) start and manage jobs, (4) monitor currently running jobs, and (5) request the imputed data. This spares the user the need to use the command line and familiarize themselves with the parameters of the scripts. In addition, this automation of job submission and management would minimize the risk of user-caused errors in the workflow.

Furthermore, the service can be connected to API-based data sources, which allow the data set to be pre-filtered by requesting only a small slice rather than the entire data set. By making several requests, either a merged VCF file can be generated, or the individual regions can be stored as independent VCF files. This would depend on the further processing. With DivBrowse, a BrAPI endpoint is offered for genotypic data, as described before. In order to be able to use this source, a consumer [42] is developed that allows individual regions to be requested and converted into VCF files, which are then imputed. This consumer can be generic so that it can be used in the web interface for data submission.

### 4.2 Improvements to imputation

Bottleneck for the imputation on a single node is the construction of the HMM for the regions on the dataset. By running the imputation as described in the previous section, those regions get imputed sequentially without any parallelization due to hardware limitations. However, by simply extending the environment to a multi-node setup and running independent imputation jobs for the different chromosomes in parallel, the run-time will decrease significantly. Furthermore, it is possible to run independent jobs in parallel for genomic windows on the chromosomes based on the identical by descent (IBD) segments that can be identified beforehand, decreasing the run-time even more. In DivImpute, the parallelization by chromosome-based job batches is already available, the imputation on genomic windows is yet to be implemented.

**Figure 3:**
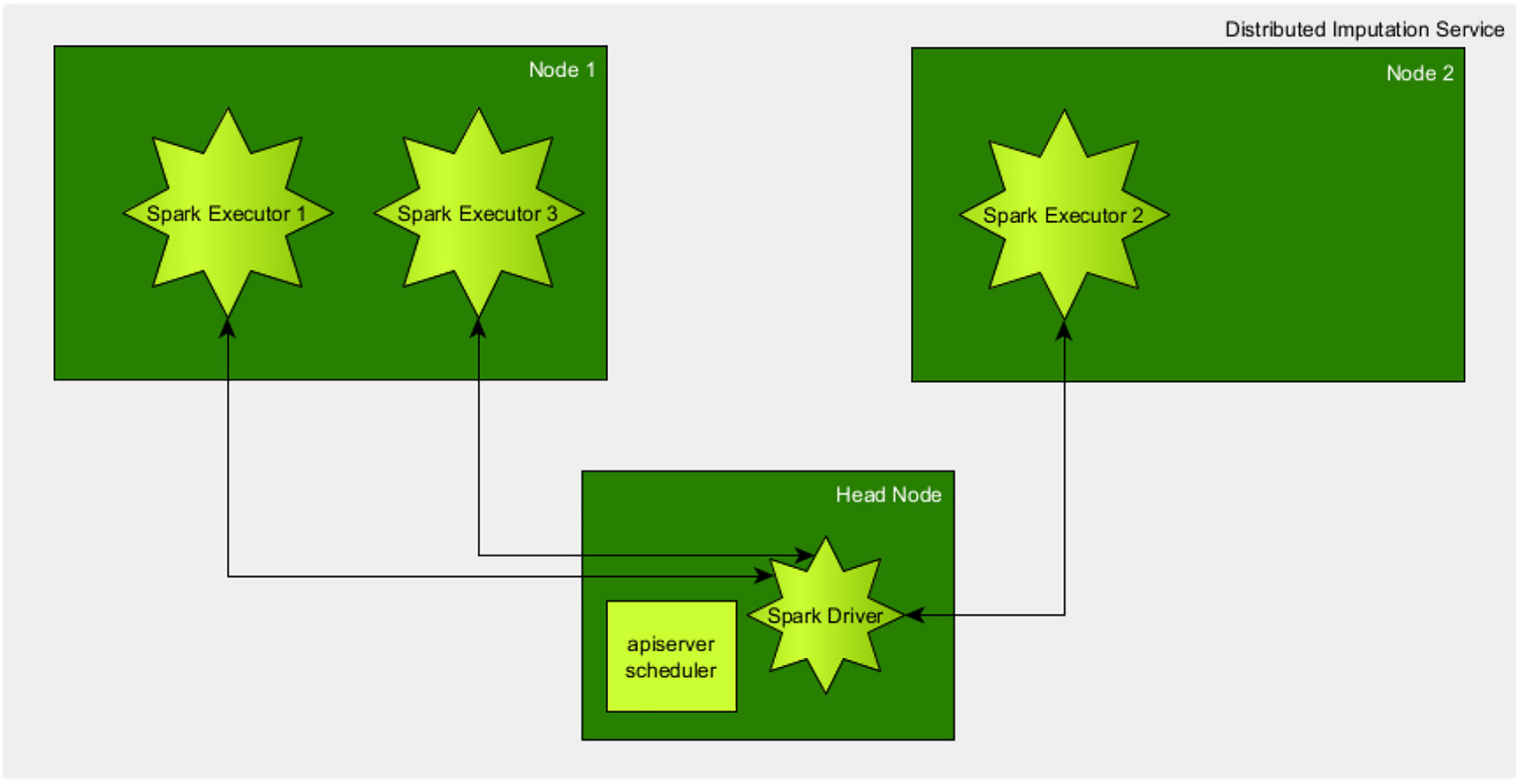
Depiction of the Architecture of Spark running in kubernetes. Head node creates a Pod for the Spark driver and schedules Spark executors on the computational nodes which are placed based on some load balancing. Example shown for only two computational nodes, in the real infrastructure multiple computational nodes would be added to the kubernetes cluster. Roughly inspired by https://spark.apache.org/docs/latest/img/k8s-cluster-mode.png.

To reduce the work the user has to put in to start the imputation, one can decide to use workflow languages like Common Workflow Language (CWL) [43] or nextflow.io [44]. Those allow to automate the scripts and reduce user input significantly. Another option is to containerize the service and run on a Kubernetes cluster. Kubernetes allows the automated deployment of containerized applications and helps in orchestrating the different jobs. Additionally, one can opt to introduce Apache Spark, which is an analytics engine for large-scale data processing, providing interfaces for Java, Scala, Python and R. With version 2.3 the support for deployment of Spark in Kubernetes was introduced. This allows running Spark jobs inside a Kubernetes Cluster by the creation of a Spark driver, which starts the independent jobs as Spark executors, collecting the results of all of those jobs back in the driver and terminating the executors when finished (cf. Figure 3). In addition, such an approach via job orchestration platforms allows imputations on different data sets to be processed in parallel in the same system, as Kubernetes ensures that the loads are balanced between the nodes of the cluster and that individual nodes do not have to wait for others to finish as well as encapsulating the data to the job instead of to the node.

With SparkBeagle [45] there exists a ready-to-use solution utilizing Spark running on Hadoop with a Hadoop Distributed File System (HDFS) set up for enabling an efficient parallel data distribution. Besides those system requirements, the concept of this solution is close to the described improvements. Instead of parallelizing the HMM algorithm, it utilizes the original Beagle version on chromosomal regions with overlap, only using the results of those markers that are at the end of a region in those overlaps.

### 4.3 Test run results

To test the procedure, the imputation of the test data set from the previous section was repeated. This time, however, the environment was extended by 2 additional virtual machines. Firstly, a head node was created, which is smaller in specification and is only used to access the computational nodes and manage the jobs. This node has 4 VCPUs, 4 GB RAM and 20 GB disk as well as 2 TB additional volume storage. This head node has access to 2 computational nodes. Both of these nodes are identical and have the same specifications as before in the single node test run.

In order to start the imputation, the data set was first divided according to chromosomes in the head node and then distributed batch wise to the computational nodes. The batches are based on the three sub genomes of wheat, so that the A, B and D genomes were imputed separately. The A and B genomes were imputed in parallel, and the D genome as soon as one node finished imputing either the A or B genome. The A genome was imputed in almost 128 h and the B genome in almost 182 h. When the imputation of the A genome was finished, the D genome jobs were started, taking roughly 69 h. The significant differences in imputation time for the three sub genomes can easily be explained by taking a look at the number of markers for the different chromosomes. Comparing the number of markers for chromosome 7 of all sub genomes reveals that with 58k markers the D genome has almost half the number of markers of A (ca. 101k) and B (ca. 110k) genomes (cf. Table 2). The test run took in total a time of 196 h (cf. Table 3 for a more detailed look on the computation times). With the introduction of an additional computational node, this could be reduced to the time for imputing the B genome. Obviously, this effect could be stacked up by introducing additional nodes and splitting the sets further.

**Table 2:** Marker numbers for the test data set over the chromosomes.

Batch/Sub genome	1	2	3	4	5	6	7	Un
A	68.585	87.175	69.937	82.485	66.145	60.659	101.576	24.328
B	111.531	128.928	125.726	70.935	93.877	119.334	109.959	
D	39.915	54.446	47.245	29.754	39.267	36.708	58.754	

**Table 3:** Tables with Run times for the different runs over batches of chromosomes on multiple nodes. Test run was executed on two identical virtual machines with 14VCPU and 128 GB RAM. The first two batches (A and B genomes) were submitted to the nodes and the third batch (D genome) submitted as soon as the first of both was completed. The A genome batch also included the SNPs of unknown chromosomes. With three nodes available this is not necessary, so for the total run time this is neglected. Bold written Total Run Times signal, which chromosome imputation was the run time determining process.

CHROM	1A	2A	3A	4A	5A	6A	7A	Un
Total time for building model	27:41:38	28:24:45	25:11:19	30:19:36	20:54:12	18:02:21	31:43:32	7:16:16
Total time for sampling	80:52:26	84:43:07	78:13:08	85:20:40	73:55:00	74:42:16	94:15:20	33:03:18
Total run time	110:19:34	114:41:25	104:37:37	116:36:09	96:03:31	94:02:02	**127:42:43**	40:52:48
**CHROM**	**1B**	**2B**	**3B**	**4B**	**5B**	**6B**	**7B**
Total time for building model	32:52:11	38:53:45	42:29:51	17:42:14	32:26:24	41:16:28	28:22:25
Total time for sampling	137:06:07	140:36:46	133:20:52	88:57:03	125:24:27	130:20:30	137:08:53
Total run time	171:54:18	**181:54:51**	178:13:55	107:26:51	159:12:25	174:18:51	177:09:47
**CHROM**	**1D**	**2D**	**3D**	**4D**	**5D**	**6D**	**7D**
Total time for building model	09:18:28	13:31:48	11:23:20	6:12:21	11:39:41	08:22:06	11:51:05
Total time for sampling	40:33:41	54:33:20	46:35:33	35:01:10	41:39:05	36:41:32	56:50:56
Total run time	50:21:23	68:47:27	58:34:50	41:40:25	53:48:04	45:29:34	**69:07:47**

### 4.4 Considerations

In summary, some advantages and disadvantages can be derived for the described concept. In addition to the improvements in usability, the acceleration or the reduction of the runtime of the imputation is a main advantage. Moreover, this is expected to happen without a loss of accuracy of the results. In addition, the introduction of modern infrastructure solutions such as Spark and Kubernetes allow jobs to be automated and the workload to be distributed among the computational nodes. This can reduce the runtime even further. By running ready-to-use solutions such as SparkBeagle, the management of merging the individual partial results, as well as cleaning up the executor is implemented in a fault tolerant way. In general fault tolerance is ensured by the usage of for example Kubernetes, as pods will be rescheduled, when a node is lost. In the current solution a node fault will result in the processes to be lost and need to be started again manually.

However, by using ready-to-use solutions, one is less flexible and limited in the decisions and customization of the system. SparkBeagle requires Hadoop as well as Hadoop Distributed File System. Therefore, one would need to deploy as well as maintain those additional systems. Besides technical requirements, such solutions also limit the usage of the imputation software, as SparkBeagle seems, based on the documentation, to not support imputation solely based on the target set, but only based on a reference panel. Therefore, reducing the flexibility of the service. Furthermore, it seems the active development of SparkBeagle has been stopped, showing – with stopped maintenance – another disadvantage of ready-to-use solutions. Apart from using ready-to-use solutions, the introduction of Kubernetes and Spark require one to coordinate the complex interplay of the additional components, maintain their functionality as well as introduce security policies on those systems. One way of dealing with this, is to rely on specialized services providing such environments in a platform-as-a-service (PAAS) style, which allow for an easier and faster deployment of such an imputation service.

## 5 Summary and participation in research networks

In this article, a use case for the analysis of genotyping data in the field of crop plant research has been described. Here, the transfer of analyses pipelines from local, institutional IT systems to external distributed infrastructures has been presented. As an exemplary application, the research field of genotyping with the subfield of imputation was considered. For an research institute like the IPK Gatersleben, which holds over 150,000 different samples of cultivated plants in its genebank, their development and activation is an important task and challenge in order to further develop the IPK Genebank in particular as well as the whole IPK in general into a bio-digital resource center. Currently, these genebank samples are systematically genotyped in many projects including following publication of the results in scientific articles and the provision of the data sets regarding the FAIR criteria in international repositories, in information systems hosted at IPK and accessible via the web as well as comprehensive data publications. Further analysis and integration of this published information with datasets from interesting users in the scientific community is currently one of the greatest challenges. Thus, the easy reuse of the research data is currently in the focus.

Hence, the availability of research data that meets the FAIR criteria is thus increasingly fulfilled. This is achieved by consistently applying metadata standards for description, by offering the data in standard formats, and by providing access via standardized APIs. But how can reuse of the research data be further advanced? The current task is to build up infrastructures, offer them sustainably and operate them permanently. A single research institute cannot fulfill this task alone, because the necessary resources are not available locally or cannot be acquired.

In the area of life sciences, a decentralized, federated infrastructure was established with the mentioned de.NBI network, which is currently being sustained. One component of de.NBI is the de.NBI cloud, which allows the scientific community to perform its analyses in data centers distributed all over Germany. Thereby, the offered compute resources can be used with publicly available as well as own data. The de.NBI cloud was used to compute the imputation described in the use case in this present article.

It was recognized that the development to build sustainable infrastructures needs to go further. In Germany, for example, the National Research Data Infrastructure (NFDI) has been under establishment since 2020. Here, nearly all scientific domains far beyond the life sciences are working together to offer sustainable solutions so that research data can be published in accordance with the FAIR criteria, remain accessible and also be reused. Through this collaboration across all scientific disciplines, it is expected to exploit appropriate synergies in building and operating the infrastructures to achieve sustainability more effectively. This concept is called the Research Data Commons (RDC).

The IPK Gatersleben is contributing actively to the NFDI in the consortia NFDI4Biodiversity and FAIRagro and is working together with NFDI collaborators to deploy and sustain the imputation use case described in this article into the NFDI RDC infrastructure as one part of Plant Data Commons.
